# Long-Term Outcomes of Endoscopic Gallbladder Drainage for Cholecystitis in Poor Surgical Candidates: An Updated Comprehensive Review

**DOI:** 10.3390/jcm10214842

**Published:** 2021-10-21

**Authors:** Tadahisa Inoue, Michihiro Yoshida, Yuta Suzuki, Rena Kitano, Fumihiro Okumura, Itaru Naitoh

**Affiliations:** 1 Department of Gastroenterology, Aichi Medical University, 1-1 Yazakokarimata, Nagakute 480-1195, Japan; kitano.rena.035@mail.aichi-med-u.ac.jp; 2 Department of Gastroenterology and Metabolism, Graduate School of Medical Sciences, Nagoya City University, 1 Kawasumi, Mizuho-cho, Mizuho-ku, Nagoya 467-8601, Japan; mityoshi@med.nagoya-cu.ac.jp (M.Y.); inaito@med.nagoya-cu.ac.jp (I.N.); 3 Department of Gastroenterology, Gifu Prefectural Tajimi Hospital, 5-161 Maehata-cho, Tajimi 507-8522, Japan; suzuki-yuta@tajimi-hospital.jp (Y.S.); okumura-fumihiro@tajimi-hospital.jp (F.O.)

**Keywords:** acute cholecystitis, recurrent cholecystitis, endoscopic gallbladder stenting, endoscopic ultrasound-guided gallbladder drainage

## Abstract

Laparoscopic cholecystectomy is the standard and fundamental treatment of choice for acute cholecystitis; however, there are cases in which patients may be poor surgical candidates due to advanced age, comorbidities, and/or general condition. The rate of recurrent cholecystitis is high in patients who are not surgically treated; therefore, the prevention of recurrence in this patient population is an important subject of investigation in the management of cholecystitis. Although it has recently been reported that long-term stent placement by endoscopic gallbladder stenting or endoscopic ultrasound-guided gallbladder drainage may reduce the recurrence rate, its efficacy and safety remain controversial. Additionally, details surrounding the long-term stent management of these treatment methods should be further investigated. In this review, we summarize the updated evidence regarding the usefulness of long-term stent placement with endoscopic gallbladder stenting or endoscopic ultrasound-guided gallbladder drainage as a preventive measure for recurrence of cholecystitis and discuss issues that should be addressed in future studies.

## 1. Introduction

Acute cholecystitis is a very common condition wherein approximately 90% of cases are caused by gallbladder stones [1,2]. The main pathogenic mechanisms of acute cholecystitis are cystic duct obstruction due to the impaction of the stones and intracholecystic cholestasis. Early cholecystectomy is the standard and definitive treatment of choice for acute cholecystitis, but patients who are unsuitable for emergency cholecystectomy are initially managed with gallbladder decompression [3,4]. There are two main approaches to gallbladder decompression [5]: percutaneous and endoscopic ones. The percutaneous approach includes percutaneous transhepatic gallbladder aspiration (PTGBA) and percutaneous transhepatic gallbladder drainage (PTGBD). The endoscopic approach includes endoscopic naso-gallbladder drainage (ENGBD), endoscopic gallbladder stenting (EGBS), and endoscopic ultrasound-guided gallbladder drainage (EUS-GBD). In recent years, the implementation of endoscopic drainage has been increasing with the progress of techniques and the advancement of devices. Additionally, EGBS and EUS-GBD avoid the use of external drainage catheters and thus provide a benefit to patient quality of life and obviate the risk to self-remove the drainage tubes. After achieving infection resolution and clinical improvement following initial gallbladder decompression, elective cholecystectomy is recommended to prevent recurrence [3].

However, there are cases where surgery is difficult or not indicated even in an elective setting after drainage and the improvement of infection due to the patient’s advanced age and/or underlying disease. Recurrent cholecystitis frequently occurs if cholecystectomy is not performed in acute cholecystitis; the reported recurrence rate ranges from 22 to 47% in patients who did not undergo cholecystectomy after percutaneous gallbladder drainage [6,7,8]. These patients can experience frequent, repeated acute cholecystitis; therefore, the long-term management of cholecystitis in poor surgical candidates of cholecystectomy is a major concern. Recently, it has been suggested that long-term stent placement by EGBS or EUS-GBD may reduce the recurrence rate of cholecystitis. However, there is no clear consensus yet, and no detailed review article to date has explored the current state of knowledge pertaining to this subject. In this comprehensive narrative review, we provide an updated summary of the current evidence found on the PubMed database while discussing existing controversies and future prospects of the use of EGBS or EUS-GBD as a preventive measure for recurrent cholecystitis in poor surgical candidates.

## 2. EGBS vs. PTGBD for Long-Term Outcomes

**Figure 1:**
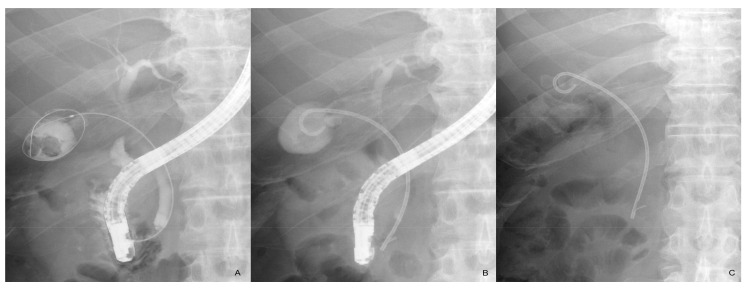
Endoscopic gallbladder stenting. The cystic duct is sought with a guidewire after biliary cannulation and the guidewire is inserted and placed in the gallbladder (**A**). A 7 Fr pigtail plastic stent is placed from the gallbladder to the duodenum (**B**). The stent remains in place 2 years after the procedure with no recurrent cholecystitis (**C**).

ENGBD and EGBS are classified as transpapillary approaches; a naso-gallbladder tube is placed in ENGBD and a plastic stent extending from the gallbladder to the duodenum is placed in EGBS [9]. Since EGBS is an internal fistula method, the tube can be indwelling for a long period of time without impairing a patient’s quality of life; in fact, long-term placement can be useful in preventing cholecystitis recurrence in patients with end-stage liver disease [10,11,12] and poor surgical candidates [13,14,15,16,17,18,19,20,21,22,23,24,25,26] (Figure 1). Based on research surrounding biliary stent placement for malignant or benign biliary strictures, it is unlikely that the stent will remain patent for years [27,28]. However, it appears that the stent not only facilitates bile drainage but also prevents gallstone impaction, thereby preventing recurrent cholecystitis. Additionally, even if stent occlusion were to occur, “wicking”, which causes bile to flow along the outer surface of the stent, may effectively prevent recurrence [29].

To date, three retrospective comparative studies [30,31,32] have investigated the usefulness of long-term stent placement via EGBS in poor surgical candidates with acute cholecystitis (Table 1). Kedia et al. [30] compared outcomes between patients who underwent EGBS (the study also includes some cases of EUS-GBD) and patients who underwent PTGBD followed by removal of the tube after clinical improvement. They reported that the mean durations of follow-up for each cohort were 9.4 months in the percutaneous drainage group and 8.8 months in the endoscopic drainage group (*p* = 0.38), and significantly more late adverse events, including recurrent cholecystitis, occurred in the percutaneous drainage group (27.9% vs. 0%, *p* < 0.0001). Inoue et al. [31] compared patients who underwent observation with tube removal after percutaneous drainage and those who underwent EGBS. Stents were kept without any stent exchange in the EGBS group. The median duration of follow-up was 485 days in the observation group and 473 days in the EGBS group, with no significant difference (*p* = 0.649). The recurrence rate of cholecystitis was significantly higher in the observation group (17.2% vs. 0%, *p* = 0.043), but the rate of overall biliary events, which not only include cholecystitis but also cholangitis, was not significantly different (24.1% vs. 9.1%, *p* = 0.207). Maruta et al. [32] compared the outcomes of patients with the removal of the gallbladder drainage tube after PTGBD or ENGBD and those with long-term stent placement by EGBS. Both the cumulative cholecystitis recurrence rate (16.0% vs. 5.0%, *p* = 0.024) and the cumulative late adverse event rate (22.1% vs. 5.0%, *p* = 0.002) were significantly higher in the removal group than in the EGBS group, with median follow-up periods of 307 days and 375 days, respectively (*p* = 0.577).

Based on the results of the three studies, long-term stent placement with EGBS is expected to have a preventive effect on the recurrence of cholecystitis, but the results were inconclusive regarding the overall adverse event rate such as cholangitis. There are also reported cases of stent–stone complex formation instigated by the presence of a biliary stent in the bile duct for an extended period, and cases of liver abscesses caused by cholestasis in the bile duct [18]. Therefore, the possibility of increased rates of adverse events other than cholecystitis, such as cholangitis and liver abscess formation with long-term stent placement, cannot be ruled out and warrants future investigation. In addition, there have been reports of rare side effects, such as a case of a migrated stent blocking the pancreatic duct orifice and causing pancreatitis [33] and a case of gallbladder perforation due to long-term contact with the stent [34]. Given that the median or mean observation period only lasts approximately 1 year in all the studies to date, the long-term safety and efficacy of stent placement are still unclear. In cases of extremely prolonged stent placement, it may be beneficial to replace or remove it as appropriate.

Furthermore, EGBS is a more technically difficult procedure than PTGBD; in fact, in previous studies, some patients underwent PTGBD after EGBS failed. The presence of cystic duct stones, dilatation of the common bile duct, and direction of the cystic duct were reported as risk factors affecting technical failure [35]. Although the success rate of the procedure has been increasing in recent years, owing to advances in both the procedural devices and techniques, the success rate remains at approximately 75–94.1% even in recent studies presenting results of cholangioscopic guidance by experienced endoscopists [36,37]. Moreover, patients need to be under conscious sedation for EGBS compared to local anesthesia for PTGBD, and the nature of early adverse events in EGBS and PTGBD are significantly different. There is a concern that the events associated with EGBS, such as pancreatitis, may be more severe than those associated with PTGBD. To establish and implement more widespread use of this treatment method, improving the success rate is crucial, and it is also necessary to verify whether the severity of early adverse events does not increase. In any case, given that there are only three retrospective studies to date, which were limited by selection and publication bias, further randomized controlled trials (RCT) are necessary.

## 3. EUS-GBD vs. PTGBD for Long-Term Outcomes

**Figure 2:**
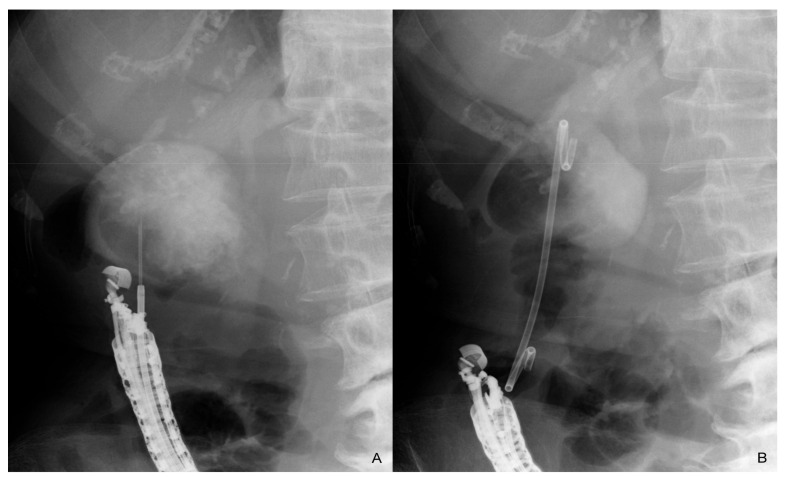
Endoscopic ultrasound-guided gallbladder drainage. After the gallbladder is punctured transduodenally (**A**), a guidewire is placed in the gallbladder. A 7 Fr double-pigtail plastic stent is placed after dilation of the fistula (**B**). The stent remains in place after the procedure without any stent exchange and removal.

EUS-GBD is a procedure that involves puncturing the gallbladder transgastrically or transduodenally under EUS guidance to place a naso-gallbladder drainage tube, double-pigtail plastic stent, or metal stent [38]. There are some specialized metal stents for use in EUS-GBD such as a metal stent with an anti-migration system, but there has recently been an increasing number of reports showing the usefulness of the lumen-apposing metal stent (LAMS) for EUS-GBD [39]. It is also suggested that long-term stent placement by EUS-GBD prevents the recurrence of cholecystitis [40,41,42,43,44,45] (Figure 2).

Three retrospective studies [46,47,48] and one RCT [49] have compared EUS-GBD and PTGBD and described the long-term outcomes of these methods (Table 2). Irani et al. [46] conducted a retrospective study to compare EUS-GBD using LAMS vs. PTGBD. Although there was no significant difference in the rate of adverse events, including cholecystitis recurrence, the EUS-GBD group had fewer repeat interventions (*p* = 0.001). Tyberg et al. [47] similarly reported that there was a significantly higher number of patients requiring repeat interventions in the percutaneous drainage group compared with the EUS-GBD group (27.78% vs. 9.52%, *p* = 0.037). However, in these two reports, nearly half of the study participants’ cholecystitis was associated with a malignant biliary stricture, and the median or average observation period was brief, lasting less than 1 year. Their results should be interpreted in consideration of these limitations. Prognosis tends to be poor for cholecystitis associated with unresectable pancreato-biliary malignancy, and the time course of cholecystitis and long-term recurrence prevention may not be a priority in the care of these patients.

Teoh et al. [48] conducted a retrospective comparative study that only examined calculous cholecystitis. They mentioned that although the rate of recurrent acute cholecystitis was similar between the percutaneous and EUS-GBD groups (6.8% vs. 0%, *p* = 0.12), the overall adverse event rates were significantly higher in patients who underwent percutaneous cholecystostomy (74.6% vs. 32.2%, *p* < 0.001). However, it should be noted that in the study, the mean duration of follow-up was 834.1 days in the percutaneous group and 450.7 days in the EUS-GBD group, showing a significant difference (*p* < 0.001). Teoh et al. later conducted an RCT [49] comparing EUS-GBD using LAMS and PTGBD in patients with calculous cholecystitis, as in their prior retrospective study. Patients who received EUS-GBD were scheduled for a follow-up for oral cholecystoscopy via the LAMS one month after the procedure, and if all gallstones were cleared, the LAMS was removed and replaced with a permanent 7 Fr double-pigtail plastic stent. All patients were followed-up for 1 year or until death. Significantly fewer patients in the EUS-GBD group had recurrent acute cholecystitis (20% vs. 2.6%, *p* = 0.029), and EUS-GBD significantly reduced adverse events by 1 year following the procedure (77.5% vs. 25.6%, *p* < 0.001). However, the total rate of recurrent biliary complication at 1 year was 20% in the PTGBD group and 10.3% in the EUS-GBD group, which was not statistically significant (*p* = 0.227).

From these research results, it can be said that long-term stent placement by EUS-GBD may be useful for reducing recurrent cholecystitis and further biliary events. However, there is no clear consensus yet in the existing literature. Even if EUS-GBD is useful for preventing cholecystitis recurrence, it is unclear whether LAMS/metal stents should be left to indwell for a long period of time, replaced with a plastic stent, or removed after symptom improvement. Long-term placement of LAMS can cause buried LAMS syndrome [41]. With the exception of cholecystitis associated with unresectable malignant biliary strictures, that is, as long as long-term survival is expected, it may be better to remove LAMS with or without plastic stent replacement. Alternatively, if a long-term placement is planned, the initial placement of a plastic stent may be an option. It is also unknown whether gallstone removal should be attempted when the stent is removed and replaced, although one retrospective study reported that EUS-GBD followed by the removal of gallstones had a rate of recurrent biliary events comparable to laparoscopic cholecystectomy, based on their one-year follow-up data [50]. More robust evidence regarding the utility and implications of EUS-GBD in preventing recurrent cholecystitis is necessary, and further long-term observation is warranted.

## 4. EGBS vs. EUS-GBD for Long-Term Outcomes

As mentioned above, long-term stent placement with EGBS and EUS-GBD are both considered treatment methods with the potential for preventing the recurrence of cholecystitis. Two retrospective studies [51,52] comparing the long-term outcomes of EGBS and EUS-GBD have been reported (Table 3). One was a study by Oh et al. [51], in which a 7 Fr double-pigtail stent was used for EGBS, and a covered metal stent was used for EUS-GBD. In both cases, patients were followed up without regular stent exchange or stent removal. After adjustment with the inverse probability of treatment weighting, both technical success (86.6% vs. 99.3%, *p* < 0.01) and clinical success (86.0% vs. 99.3%, *p* < 0.01) were significantly higher in the EUS-GBD group, while the procedure-related adverse event rate (19.3% vs. 7.1%, *p* = 0.02) was significantly lower in the EUS-GBD group. Regarding long-term outcomes, the recurrence rates of cholecystitis or cholangitis were 12.4% and 3.2% in the EGBS group and the EUS-GBD group, respectively, reflecting a significant difference (*p* = 0.04), with the mean follow-up periods of 20.7 months and 21.9 months, respectively (*p* = 0.06). Another study reported by Higa et al. [52] compared EGBS that used a 7 Fr double-pigtail stent and EUS-GBD that used LAMS. Clinical success rate was significantly higher in the EUS-GBD group (76.3% vs. 95.0, *p* = 0.020), and recurrent cholecystitis rate was lower in the EUS-GBD group (18.8% vs. 2.6%, *p* = 0.023). However, in the study, the median follow-up period was as short as 5 months in the EGBS group and 7 months in the EUS-GBD group, and 56.2% of the patients in the EGBS group and 10.3% in the EUS-GBD group eventually underwent surgical cholecystectomy. Therefore, it seems to be a slightly different study from the viewpoint of the usefulness of long-term stent placement for preventing recurrence. As a further note, both studies involved a considerable number of patients with cholecystitis associated with malignant biliary stricture with/without biliary stent placement.

Based on the results of available studies, EUS-GBD may be superior in terms of technical and clinical success, as well as in preventing recurrence, compared with EGBS. However, the fistula formation by EUS-GBD may have a negative effect if elective cholecystectomy becomes possible later (this has not been fully investigated yet). Moreover, calculous cholecystitis and cholecystitis associated with malignant biliary stricture differ in multiple aspects, including pathogenic mechanisms, long-term course, and treatment strategies. These should be considered separately, especially when considering long-term outcomes, including recurrence, which has not been done in studies to date. It is considered that the prevention of recurrent cholecystitis is more important in calculous cholecystitis cases and less so in cases of advanced malignancy with limited prognoses. Consequently, it will be difficult to directly apply the results of current studies to the long-term management of patients with cholecystitis who are poor surgical candidates for cholecystectomy. Future studies that compare the results of treatment via EGBS vs. EUS-GBD through better-controlled, standardized study designs may further help this field of research.

## 5. Conclusions

In this review article, we discussed the current state of knowledge, shortcomings, and prospects of endoscopic management for preventing recurrent cholecystitis in patients unfit for cholecystectomy. It is particularly important to prevent recurrence in this patient population. Long-term stent placement with EGBS and EUS-GBD is a therapeutic method that may be a useful option for the prevention of recurrent cholecystitis. It is expected that the efficacy and safety of these procedures will be better established by future studies.

## Figures and Tables

**Table 1:** Studies comparing long-term outcomes of endoscopic gallbladder stenting and percutaneous drainage.

Author	Study Design	Drainage Method	No. of Patients	Drainage Tube/Stent	Technical Success		Clinical Success		Early Adverse Event		Follow-Up Period (Median/Mean)		Recurrent Cholecystitis		Late Adverse Event (Including Recurrent Cholecystitis)	
Kedia et al., 2015 [30]	Retrospective	EGBS ^†^	30 ^†^	5 or 7 Fr pigtail	100%	*p* = 0.58	86.7%	*p* = 0.08	13.3%	*p* = 0.55	8.8 m	*p* = 0.39	-	-	0	*p* < 0.0001
		PTGBD	43	8 or 10 Fr	97.6%		97.6%		11.6%		9.4 m		-		27.9%	
Inoue et al., 2016 [31]	Retrospective	EGBS	35	7 Fr pigtail	82.9%	-	82.9%	-	2.9%	-	15.6 m	*p* = 0.649	0	*p* = 0.043	9.1%	*p* = 0.207
		PTGBD/PTGBA	29	PTGBD: 7 or 8.5 Fr	-		-		-		16.0 m		17.2%		24.1%	
Maruta et al., 2021 [32]	Retrospective	EGBS	40	5 or 6 Fr pigtail	78.9% ^‡^	*p* < 0.0001	94.6% ^‡^	*p* = 1.000	4.2% ^‡^	*p* = 1.000	12.3 m	*p* = 0.577	5.0%	*p* = 0.024	5.0%	*p* = 0.002
		PTGBD/ENGBD	131	PTGBD: 8 or 8.5 Fr ENGBD: 5 or 6 Fr	100% ^‡^		93.5% ^‡^		4.5% ^‡^		10.1 m		16.0%		22.1%	

EGBS, endoscopic gallbladder stenting; PTGBD, percutaneous transhepatic gallbladder drainage; PTGBA, percutaneous transhepatic gallbladder aspiration; ENGBD, endoscopic naso-gallbladder drainage. ^†^ Some cases of endoscopic ultrasound-guided gallbladder drainage were included. ^‡^ EGBS/ENGBD vs. PTGBD.

**Table 2:** Studies comparing long-term outcomes of endoscopic ultrasound-guided gallbladder drainage and percutaneous drainage.

Author	Study Design	Drainage Method	No. of Patients	Drainage Tube/Stent	Technical Success		Clinical Success		Early Adverse Event		Follow-Up Period (Median/Mean)		Recurrent Cholecystitis		Late Adverse Event (Including Recurrent Cholecystitis)	
Irani et al., 2017 [46]	Retrospective	EUS-GBD	45	LAMS	98%	*p* = 0.98	96%	*p* = 0.12	18% ^†^	*p* = 0.07	7.1 m	*p* = 0.25	6.7%	-	-	-
		PTGBDf	45	8 or 10 Fr	100%		91%		31% ^†^		8.7 m		8.9%		-	
Teoh et al., 2017 [48]	Retrospective	EUS-GBD	59	LAMS	96.6%	*p* = 0.15	89.8%	*p* = 0.30	28.8%	*p* = 0.13	14.9 m	*p* < 0.001	0	*p* = 0.12	32.2% ^‡^	*p* < 0.001
		PTGBD	59	6–10 Fr	100%		94.9%		16.9%		27.5 m		6.8%		74.6% ^‡^	
Tyberg et al., 2018 [47]	Retrospective	EUS-GBD	42	PS/CSEMS/LAMS	95.23%	*p* = 0.179	95.23%	*p* = 0.157	4.76%	*p* = 0.613	4.4 m	-	7.1%	-	16.67%	*p* = 0.783
		PTGBD	113	-	99.12%		88.18%		2.65%		7.6 m		8.0%		18.58%	
Teoh et al., 2020 [49]	RCT	EUS-GBD	39	LAMS	97.4%	*p* = 0.494	92.3%	*p* = 1	12.8%	*p* = 0.001	- ^§^	-	2.6%	*p* = 0.029	10.3%	*p* = 0.227
		PTGBD	40	8.5 Fr	100%		92.5%		47.5%		- ^§^		20%		20%	

RCT, randomized controlled trial; EUS-GBD, endoscopic ultrasound-guided gallbladder drainage; PTGBD, percutaneous transhepatic gallbladder drainage; LAMS, lumen-apposing metal stent; PS, plastic stent; CSEMS, covered self-expandable metal stent. ^†^ Late adverse events were also included. ^‡^ Early adverse events were also included. ^§^ Patients were followed-up until one year or death.

**Table 3:** Studies comparing long-term outcomes of endoscopic gallbladder stenting and endoscopic ultrasound-guided gallbladder drainage.

Author	Study Design	Drainage Method	No. of Patients	Stent	Technical Success		Clinical Success		Early Adverse Event		Follow-Up Period (Median/Mean)		Recurrent Cholecystitis		Late Adverse Event (Including Recurrent Cholecystitis)	
Oh et al., 2019 [51]	Retrospective	EGBS	96	7 Fr pigtail	86.6% ^†^	*p* < 0.01	86.0% ^†^	*p* < 0.01	19.3% ^†^	*p* = 0.02	20.7 m ^†^	*p* = 0.73	10.5% ^†^	-	12.4% ^†^	*p* = 0.04
		EUS-GBD	83	CSEMS	99.3% ^†^		99.3% ^†^		7.1% ^†^		21.9 m ^†^		3.2% ^†^		3.2% ^†^	
Higa et al., 2019 [52]	Retrospective	EGBS	38	7 Fr pigtail	84.2%	*p* = 0.072	76.3%	*p* = 0.020	9.4% ^‡^	*p* = 0.80	5 m	*p* = 0.80	18.8%	*p* = 0.023	-	-
		EUS-GBD	40	LAMS	97.5%		95.0%		17.9% ^‡^		7 m		2.6%		-	

EGBS, endoscopic gallbladder stenting; EUS-GBD, endoscopic ultrasound-guided gallbladder drainage; CSEMS, covered self-expandable metal stent; LAMS, lumen-apposing metal stent. ^†^ These were evaluated using inverse probability of treatment weighting. ^‡^ Late adverse events were also included.
